# Direction-Of-Arrival Estimation and Tracking Based on a Sequential Implementation of C-SPICE with an Off-Grid Model

**DOI:** 10.3390/s17122718

**Published:** 2017-11-24

**Authors:** Shu Cai, Xiaoye Shi, Hongbo Zhu

**Affiliations:** 1 Jiangsu Key Laboratory of Wireless Communication, Nanjing University of Posts and Telecommunications, Nanjing 210003, China; zhuhb@njupt.edu.cn; 2 School of Internet of things, Nanjing University of Posts and Telecommunications, Nanjing 210003, China; shixy187@njupt.edu.cn

**Keywords:** DoA estimation, target tracking, sparse covariance fitting criterion, off-grid model

## Abstract

This paper focuses on the problem of estimating and tracking time-varying direction-of-arrivals (DoAs) with an antenna array. A sequential DoA estimation method is proposed by extending the capon and sparse iterative covariance-based estimation (C-SPICE) method, which is an iterative off-grid method for estimating constant DoAs. Then, a moving average initialization technique is introduced such that the spatial spectrum information estimated in this snapshot can be utilized in the next one. In uniform linear arrays (ULAs), we replace the uniform grid in direction domain with that in a “frequency” domain, to improve estimation accuracy without additional complexity in practical applications. The validity and efficiency of the proposed methods are demonstrated through numerical experiments.

## 1. Introduction

Tracking directions of multiple moving targets by antenna arrays has widely applications in both military and civilian fields, such as surveillance, air traffic control, and wireless communication [1,2]. In recent two decades, many direction-of-arrival (DoA) tracking algorithms have been proposed. For example, the subspace-based methods (see [3] and references therein), methods for wideband signals tracking (see [4] and references therein), and methods for two-dimensional direction tracking (see [5] and references therein). Comparing with DoA estimation, tracking algorithms need not only to estimate DoAs repeatedly and sequentially over time, but also to associate DoA estimates to tracks according to their sources, which is referred to as data association. Hence, many data association methods for multiple target tracking are also proposed [6,7,8,9,10].

Recently, the research on DoA estimation has been evolved due to the developments in sparse signal reconstruction (SSR) methods and many algorithms are proposed accordingly (see [11,12,13] and references therein). However, the SSR method designed for DoA tracking is rare. Some methods [14,15] can be used for DoA tracking, but they have a relatively high computational complexity. In [16], an efficient algorithm named by tracking via low-rank plus sparse recovery (TvLSR) is proposed. Different from traditional tracking algorithms, where DoAs are estimated and tracked repeatedly and sequentially over snapshots (or time windows), TvLSR estimates tracks of sources over all snapshots once for all. Hence, it is actually a block trajectory estimation method. A Bayesian sparse-plus-low-rank matrix decomposition (BSPLR) method is also proposed to improve the estimation accuracy of TvSLR [17]. These methods are based on on-grid models, whose estimation accuracy is limited by size of the grid [11]. Although an off-grid model can be incorporated, BSPLR is computationally expensive.

A capon and sparse iterative covariance-based estimation (C-SPICE) method, which estimates DoAs of stable sources based on an off-grid model, is proposed in [11] for a better estimation accuracy and computational complexity tradeoff. In this paper, we extend C-SPICE to track DoAs of moving targets by exploiting the iterative structures in it. Specifically, an efficient sequential implementation of C-SPICE is introduced to estimate DoAs in each snapshot. To save number of iterations, a moving average technique is proposed such that the spatial spectrum information estimated in this snapshot can be utilized to construct a good initial point for iterations in the next one. In uniform linear arrays (ULAs), we replace the uniform grid in direction domain by the grid in a “frequency” domain, whose density adapts automatically to the resolution ability of a ULA and is greater when DoA is small. Since the effective scanning range of a ULA may be no more than $\left(-60^{\circ },60^{\circ }\right)$ in some practical applications [1], the new grid can improve estimation accuracy without additional complexity.

*Notations:* The operators ${\left(\cdot \right)}^{T}$ and ${\left(\cdot \right)}^{H}$ denote transpose and conjugate transpose, respectively, and diag(x) returns a diagonal matrix with main diagonal x . ∥a∥ and ∥A∥ denote the $l_{2}$ -norm and Frobenius norm of a and A , respectively. $C^{N\times 1}$ represents the space of N-dimensional complex column vectors. For an integer *N*, [N] is defined as {1,2,⋯,N} . I represents an identity matrix and $1_{N}$ an N×1 vector with entries 1. Re(·) denotes real part of a complex and tr(·) calculates the trace of a matrix.

## 2. Problem Formulation

### 2.1. Signal Model

Consider the problem of estimating DoAs of *M* far-field narrow-band sources based on the outputs of an *N*-element array. A nonparametric model of the array output can be expressed by [14]:

(1) $$y\left(t\right)=\sum_{k=1}^{K}a\left({\tilde{\theta }}_{k}\right)x_{k}\left(t\right)+n\left(t\right),\;\;t\in \left[N_{t}\right],$$

where $y\left(t\right)\in C^{N\times 1}$ and n(t) denote the snapshot and the noise at time *t*, respectively, ${\left\{{\tilde{\theta }}_{k}\right\}}_{k=1}^{K}$ is a uniform and discrete grid covering the possible range of DoAs, $a\left({\tilde{\theta }}_{k}\right)\in C^{N\times 1}$ is the steering vector of ${\tilde{\theta }}_{k}$ , $x_{k}\left(t\right)\in C$ is the unknown signal waveform of a possible source at ${\tilde{\theta }}_{k}$ , and M<N≪K . Obviously, the vector $x\left(t\right)={\left[x_{1}\left(t\right),\cdots ,x_{K}\left(t\right)\right]}^{T}$ is *M*-sparse where a nonzero entry corresponds to a source.

In a tracking scenario, suppose that DoAs of sources vary continuously and slowly over neighbouring snapshots. Then the actual DoA of m-th source at time *t*, i.e., $\theta_{m}\left(t\right)$ , may not locate on but close to some grid point ${\tilde{\theta }}_{m_{k}}$ , and thus $\theta_{m}\left(t\right)={\tilde{\theta }}_{m_{k}}+\epsilon_{m_{k}}\left(t\right)$ . With this modeling mismatch, the signal model (1) can be modified into [18,19]

(2) $$y\left(t\right)=\sum_{k=1}^{K}\left[a\left({\tilde{\theta }}_{k}\right)+e\left({\tilde{\theta }}_{k}\right)\epsilon_{k}\left(t\right)\right]x_{k}\left(t\right)+n\left(t\right),$$

where $e({\tilde{\theta }}_{k})$ is the first order derivative of a(θ) at $\theta ={\tilde{\theta }}_{k}$ , $\epsilon_{k}\left(t\right)\in \left[-l_{\epsilon },l_{\epsilon }\right]$ , and $2l_{\epsilon }$ denotes the distance between neighbouring grid points. Equation (2) can be expressed in a matrix form

(3) $$\begin{array}{c} y(t)=(A+E\Phi (t))x(t)+n(t), \end{array}$$

where

(4) $$\begin{array}{cc} A & =[a\left({\tilde{\theta }}_{1}\right),a\left({\tilde{\theta }}_{2}\right),\cdots ,a\left({\tilde{\theta }}_{K}\right)], \end{array}$$

(5) $$\begin{array}{cc} E & =[e\left({\tilde{\theta }}_{1}\right),e\left({\tilde{\theta }}_{2}\right),\cdots ,e\left({\tilde{\theta }}_{K}\right)], \end{array}$$

(6) $$\begin{array}{cc} \Phi (t) & =\mathrm{diag}\left(\epsilon \left(t\right)\right),\;\;\epsilon \left(t\right)={\left[\epsilon_{1}\left(t\right),\epsilon_{2}\left(t\right),\cdots ,\epsilon_{K}\left(t\right)\right]}^{T}. \end{array}$$

### 2.2. The C-SPICE Algorithm

C-SPICE is a grid-based off-grid sparse estimation method for constant DoA estimation [11]. It enjoys an effective tradeoff between modeling accuracy and computational complexity. Here, we introduce it briefly with the time index “ (t) ” omitted for simplicity. First, the following assumptions are required in deriving the method:

1. The noise is spatially and temporally white Gaussian with covariance σI ;
2. the noise is uncorrelated with the signals;
3. the signals are uncorrelated with each other.

Note that the third assumption is not essential, but only required during the derivation [11,14]. With these assumptions, there is

(7) $$\begin{array}{cc} & R=E[yy^{H}]\\ = & \sum_{k=1}^{K}p_{k}\left(a\left({\tilde{\theta }}_{k}\right)+e\left({\tilde{\theta }}_{k}\right)\epsilon_{k}\right){\left(a\left({\tilde{\theta }}_{k}\right)+e\left({\tilde{\theta }}_{k}\right)\epsilon_{k}\right)}^{H}+\sigma I\\ = & B^{H}D^{H}PDB, \end{array}$$

where $p_{k}=E[|x_{k}{|}^{2}]$ and

(8) $$\begin{array}{cc} B & ={\left[A,E,I\right]}^{H},\;\;D=\left[\begin{array}{ccc} I & \Phi & 0\\ 0 & 0 & I \end{array}\right], \end{array}$$

(9) $$\begin{array}{cc} P & =\mathrm{diag}\left(\left[p^{T},\sigma 1_{N}^{T}\right]\right),\;\;p={\left[p_{1},p_{2},\cdots ,p_{K}\right]}^{T}. \end{array}$$

Define $\hat{R}=\sum_{t=1}^{T}y\left(t\right)y^{H}\left(t\right)/T$ . When T>N such that ${\hat{R}}^{-1}$ exists, the following optimization problem can be constructed under covariance fitting criterion [11]

(10a)minp,σ,ϵf(p,σ,ϵ)(10b)s.t.f(p,σ,ϵ)=tr(R^R−1)+tr(R^−1R),(10c)pk≥0,|ϵk|≤lϵ,∀k∈[K],(10d)σ≥0.

Problem (10) is in general nonlinear and nonconvex (see Proposition 1 in [11]) and C-SPICE is a method to approach its optimal solution. In order to make the paper self-contained, below we provide a different and new derivation of C-SPICE.

First, it is obvious that the grid size *K* is usually large and thus $|\epsilon_{k}|\leq l_{\epsilon }\ll 1$ , ∀k∈[K] . Hence, the objective function f(p,σ,ϵ) can be simplified by letting ϵ=0 in the first term, i.e., $\mathrm{tr}(\hat{R}R^{-1})$ . Then substituting (7) into (10) and omitting the terms independent of ϵ , we obtain

(11) $$\begin{array}{cc} \min_{p,\epsilon }\; & \sum_{k=1}^{K}p_{k}∥{\tilde{a}}_{k}+{\tilde{e}}_{k}\epsilon_{k}{∥}^{2},\;s.t.\;p_{k}\geq 0,\;\left|\epsilon_{k}\right|\leq l_{\epsilon },\;\forall k\in \left[K\right], \end{array}$$

where ${\tilde{a}}_{k}={\hat{R}}^{-1/2}a\left({\tilde{\theta }}_{k}\right)$ and ${\tilde{e}}_{k}={\hat{R}}^{-1/2}e\left({\tilde{\theta }}_{k}\right)$ . If $p_{k}$ is given, problem (11) is convex and $\epsilon_{k}$ can be estimated by the closed-form solution

(12) $$\begin{array}{c} {\hat{\epsilon }}_{k}=\left\{\begin{array}{cc} {\hat{\epsilon }}_{k0}, & \mathrm{if}\;\;\left|{\hat{\epsilon }}_{k0}\right|\leq l_{\epsilon },\\ -l_{\epsilon }, & \mathrm{if}\;\;{\hat{\epsilon }}_{k0}\leq -l_{\epsilon },\\ l_{\epsilon }, & \mathrm{if}\;\;{\hat{\epsilon }}_{k0}\geq l_{\epsilon }, \end{array}\right.\;\; \end{array}$$

where ϵ^k0=−Re{a˜kHe˜k}∥e˜k∥2 . In (12), it is seen that ϵ can be determined without the information of the tuple (p,σ) .

The second step estimates (p,σ) . Let $\epsilon_{k}={\hat{\epsilon }}_{k}$ and define $g_{k}=a\left({\tilde{\theta }}_{k}\right)+{\hat{\epsilon }}_{k}e\left({\tilde{\theta }}_{k}\right)$ , ∀k . Then problem (10) is simplified to

(13) $$\begin{array}{cc} \min_{p_{k}\geq 0,\forall k\in \left[K\right],\sigma \geq 0}\; & \mathrm{tr}\left(\hat{R}R_{\hat{\epsilon }}^{-1}\right)+\mathrm{tr}\left({\hat{R}}^{-1}R_{\hat{\epsilon }}\right), \end{array}$$

where $R_{\hat{\epsilon }}=\sum_{k=1}^{K}p_{k}g_{k}g_{k}^{H}+\sigma I$ . Problem (13) can be solved by SPICE. For self-containedness, a derivation of SPICE, which is different from that of [14], is provided in the Appendix.

Finally, with the estimates of ϵ and p , DoA estimates can be obtain by

(14) $$\begin{array}{c} {\hat{\theta }}_{m}={\tilde{\theta }}_{k_{m}}+{\hat{\epsilon }}_{k_{m}},\;m\in \left[M\right], \end{array}$$

where $k_{m}$ denotes the index of the *m*-th highest peak in p .

## 3. A Sequential Implementation of C-SPICE

In this section, we extend C-SPICE to the scenario of estimating moving targets in the hope of deriving a fast and accurate tracking algorithm.

### 3.1. Sequential Implementation of C-SPICE

First, we introduce a sequential implementation of C-SPICE. Suppose that DoA estimation at time *t* is based on the most recent W(≥1) snapshots. Denote Y(t)=[y(t−W+1),⋯,y(t)] and data correlation $\hat{R}\left(t\right)=Y\left(t\right)Y^{H}\left(t\right)/W$ . Based on the stochastic maximization likelihood (SML) criterion [20], DoAs at time *t* can be estimated by minimizing the following objective function

(15) $$\begin{array}{c} f_{L}\left(p,\sigma ,\epsilon \right)=\ln\left|R\right|+\mathrm{tr}\left(R^{-1}\hat{R}\left(t\right)\right). \end{array}$$

Since ln|R| is nonconvex in (p,σ,ϵ) , we approximate it by the first-order Taylor expansion at some point $R_{0}$ (which is a positive definite matrix close to the true R ) and then $f_{L}\left(p,\sigma ,\epsilon \right)$ by

(16) $$\begin{array}{c} f_{R_{0}}\left(p,\sigma ,\epsilon \right)=\mathrm{tr}\left(R_{0}^{-1}R\right)+\mathrm{tr}\left(R^{-1}\hat{R}\left(t\right)\right). \end{array}$$

If W>N , then $R_{0}=\hat{R}\left(t\right)$ is a good approximation of R and $f_{R_{0}}\left(p,\sigma ,\epsilon \right)$ becomes f(p,σ,ϵ) in (10b). So DoAs can be estimated directly by using C-SPICE. Otherwise, when W<N or source signals are highly correlated, $\hat{R}\left(t\right)$ does not coincide with the definition of R in (7). In this case, we can minimize $f_{R_{0}}\left(p,\sigma ,\epsilon \right)$ to estimate (p,σ,ϵ) with a given $R_{0}$ and then update $R_{0}$ with the estimated (p,σ,ϵ) , iteratively. This iteration is referred to as outer iteration, where $R_{0}$ can be initialized by I . In each outer iteration, $f_{R_{0}}\left(p,\sigma ,\epsilon \right)$ can be minimized by a modification of C-SPICE, in which ${\tilde{a}}_{k}=R_{0}^{-1/2}a\left({\tilde{\theta }}_{k}\right)$ and ${\tilde{e}}_{k}=R_{0}^{-1/2}e\left({\tilde{\theta }}_{k}\right)$ in Equation (12) and the iterations updating (p,σ) , i.e., Equation (A5), are modified to

(17) $$\begin{array}{c} p_{k}={\hat{p}}_{k}∥{\hat{R}}^{0.5}R_{\hat{\epsilon }}^{-1}g_{k}∥/∥R_{0}^{-0.5}g_{k}∥,\;k\in \left[K\right],\;\sigma =\hat{\sigma }∥{\hat{R}}^{0.5}R_{\hat{\epsilon }}^{-1}∥/\sqrt{\mathrm{tr}(R_{0}^{-1})}. \end{array}$$

We call the iterations updating (p,σ) by (17) inner iteration. When outer iteration converges, DoAs can be obtained by (14). We summarize the sequential implementation of C-SPICE (SIC-SPICE) in Algorithm 1, where $I_{2}$ denotes number of outer iterations in each snapshot and $I_{1}$ the number of inner iterations. If W≪N , Step 12 can be simplified by replacing ${\hat{R}}^{0.5}\left(t\right)$ with $Y^{H}\left(t\right)/\sqrt{W}$ in Equation (17), which can be readily derived by modifying ${\hat{R}}^{0.5}\left(t\right)$ to $Y^{H}\left(t\right)/\sqrt{W}$ in the constraint of (A1).

**Algorithm 1** A Sequential Implementation of C-SPICE (SIC-SPICE)1:Fix *K*, *W*, $I_{1}$ , and $I_{2}$ , and set t=W ;2:**repeat** Input Y(t) ;3:  **if** W<N and source signals are uncorrelated **then**4:    
$R_{0}=I$ and $\hat{\epsilon }=0$ ;5:  **else**6:    
$R_{0}=\hat{R}\left(t\right)$ and estimate ϵ by (12);7:  **end if**8:  **for** $i_{2}=1$ to $I_{2}$ **do**9:    Initialize (p,σ) by PER;10:    **for** $i_{1}=1$ to $I_{1}$ **do**11:       Construct R by (7);12:      Update (p,σ) by (17);13:    **end for**14:    Construct $R_{0}$ by (7);15:    Estimate ϵ by (12);16:  **end for**17:  Obtain DoA estimates by (14) ;18:  Let t=t+1 ;19:**until** $t=N_{t}$ .


Since SIC-SPICE is an iterative method, its convergence behavior needs further discussion. In each snapshot, there are two types of iterations. In inner iterations, the tuple (p,σ) is estimated with $\epsilon =\hat{\epsilon }$ . According to the derivation in Appendix, inner iterations can converge to a global optimal point (which depends on $\hat{\epsilon }$ ) from any initial value in the interior of feasible set. On the other hand, if function (16) can be minimized in each outer iteration, then the obtained optimal solutions reduce the value of $f_{L}\left(p,\sigma ,\epsilon \right)$ monotonically (see (28)–(31) in [11]). However, C-SPICE is not guaranteed to provide a point minimizing (16). In this case, we use the solution of outer iterations that minimizes $f_{L}\left(p,\sigma ,\epsilon \right)$ for DoA estimation.

The complexity of SIC-SPICE in each snapshot is $O(N^{3}KI_{1}I_{2})$ [11]. When *N* is given, we may pursuit an accuracy and complexity trade-off by choosing *K*, $I_{1}$ , and $I_{2}$ intelligently.

### 3.2. A Moving-Average Initialization Technique

To obtain an accurate estimation of (p,ϵ) , the required number of inner iterations in SIC-SPICE, i.e., $I_{1}$ , will be large, which means that a large processing time is required for each snapshot. This is undesirable, especially when the processing time is larger than the snapshot interval.

On the other hand, in the tracking scenario, one can assume that the variations of actual DoAs between neighbouring snapshots are small such that $|\theta_{m}\left(t\right)-\theta_{m}\left(t-1\right)|\leq 2Bl_{\epsilon }$ , ∀m,t , where *B* is an integer. This assumption is reasonable since time interval between neighbouring snapshots is usually tiny. Therefore, the optimal (p,σ) of this snapshot is close to that of the next one, which means that outputs of this outer iteration can be a good initial point for the next. Unfortunately, these outputs cannot be used directly to initialize (p,σ) , which can be readily observed by setting $p_{k}=0$ for some *k*. Then, according to (17), $p_{k}$ still equals zero even if a target has moved to ${\tilde{\theta }}_{k}$ . To overcome this problem, we propose a moving-average (MA) technique for initializing p and σ in each outer iteration, which is,

(18a)pk=12B+1∑l=k−Bk+Bp^l+ϵc,∀k∈[K],(18b)σ=σ^+ϵc,

where $\epsilon_{c}$ is a small number preventing $p_{k}$ and σ being zeros and *B* controls the size of the block dispersing amplitude of $p_{k}$ . Then the information obtained in previous iteration can be used and the algorithm will not converge to a trivial solution.

### 3.3. SIC-SPICE for ULAs

In a ULA, a steering vector can be expressed by $a\left(\theta \right)={\left[1,e^{j\frac{2\pi }{\lambda }d\sin\left(\theta \right)},\ldots ,e^{j\frac{2\pi }{\lambda }\left(N-1\right)d\sin\left(\theta \right)}\right]}^{T}$ , where λ and *d* denote wavelength and element spacing, respectively. Then, instead of using the uniform grid in θ -domain, i.e., ${\left\{{\tilde{\theta }}_{k}\right\}}_{k=1}^{K}$ , we use a uniform grid in the “frequency” domain, i.e., ${\left\{f_{k}\right\}}_{k=1}^{K}$ , where $f=\frac{2d}{\lambda }\sin\left(\theta \right)$ . Without loss of generality, let us assume d=λ/2 . Thus the domains of θ and *f* are $\left(-\frac{\pi }{2},\frac{\pi }{2}\right)$ and (−1,1) , respectively. By mapping ${\left\{f_{k}\right\}}_{k=1}^{K}$ into the θ -domain, the resulting grid is more fine around θ=0 and more coarse as θ approaching $\pm \frac{\pi }{2}$ . Since the resolution ability of a ULA decreases monotonically as the DoA θ approaching $\pm \frac{\pi }{2}$ [1], the density of grid in “frequency” domain adapts to resolution of the ULA automatically. Moreover, in practical applications, the effective working scope of a ULA is usually less than $\left(-\frac{\pi }{2},\frac{\pi }{2}\right)$ [1]. Hence, for a given grid size *K*, ${\left\{f_{k}\right\}}_{k=1}^{K}$ enjoys a more fine grid and thus higher estimation accuracy for DoAs close to 0.

In addition, since the correlation matrix R can be constructed based on the result of outer iterations in SIC-SPICE, subspace methods, such as multiple signal classification algorithm (MUSIC) [21], can be used to estimate DoAs in Step 17 of Algorithm 1. The advantages of subspace methods are two folds: first, estimation accuracy of the algorithm is no longer limited by the capacity of grid; second, it may avoid missing true DoAs at nearby grids, which produce only one peak.

## 4. Simulations

In this section, we evaluate the performance of the proposed algorithms by comparing them with existing SSR-based methods. The compared algorithms include SPICE [14], Algorithm 1 (SIC-SPICE), Algorithm 1 with moving average initialization (SIC-SPICE+), Algorithm 1 with moving average initialization based on uniform grid in “frequency” domain (SIC-SPICE++), tracking via low-rank plus sparse matrix decomposition (TvLSR) [16], and Bayesian sparse-plus-low-rank matrix decomposition method (BSPLR) [17]. For all SPICE kind of methods, we set W=1 such that DoAs are estimated based on the latest snapshot, apply MUSIC algorithm to estimate DoAs, and use B=1 in the moving average technique (18). In SPICE, the maximum number of iterations is 100 for the first snapshot and 6 for the others. In SIC-SPICE methods, we set $I_{1}=100$ for the first outer iteration in the first snapshot, $I_{1}=3$ for the others, and $I_{2}=2$ . All the parameters in TvLSR and BSPLR are chosen according to those in [16,17], respectively, and the maximum number of iterations for both is 150.

In all simulations, ULAs with half-wavelength spaced sensors is used, and the number of the signal sources in space is M=3 . Two kinds of trajectories are considered:

- Case-1: The first two sources move from $35^{\circ }$ to $5^{\circ }$ and from $15^{\circ }$ to $-5^{\circ }$ , respectively, over 100 snapshots. The third one moves following the function $\theta_{3}\left(t\right)=-25^{\circ }$ – $8^{\circ }\sin\left(0.05t\right)$ .
- Case-2: The trajectories of the first two sources are the same as those in Case-1. The third one moves according to $\theta_{3}\left(t\right)=-5+0.25t+5\sin\left(0.05t\right)$ .

In both cases, the signal of the *m*-th source is generated by $x_{m}\left(t\right)=\varsigma_{m}\exp\left(-j\upsilon_{m}\left(t\right)\right)$ , where $\upsilon_{m}\left(t\right)$ is uniformly distributed in [0,2π) and amplitudes have the relationship $\varsigma_{m}^{2}=3\varsigma_{m-1}^{2}$ . The signal to noise ratio (SNR) is defined by $10\log_{10}\left(\varsigma_{1}^{2}/\sigma \right)$ .

A prerequisite for multiple-target tracking is associating DoA estimates between neighbouring snapshots. This problem is nontrivial and has been discussed in many literatures (see [6,7,8,9,10] and literatures there in). Hence, we assume that the estimates are well associated in simulations. For example, we may assume that powers of different sources are different such that DoA estimates can be associated according to signal power estimation. Alternatively, in ULAs, DoA estimates can be updated by using Newton’s method to the MUSIC spectrum with estimates in the previous snapshot as initial values. Then, the estimates of neighbouring snapshots are associated automatically. In simulations, we assume that sources have different powers, i.e., $\varsigma_{m}^{2}=3\varsigma_{m-1}^{2}$ , such that data association can be easily achieved based on power estimation. This assumption is not necessary when a more powerful data association method, such as those proposed in [6,7,8,9,10] are used.

**Figure 1:**
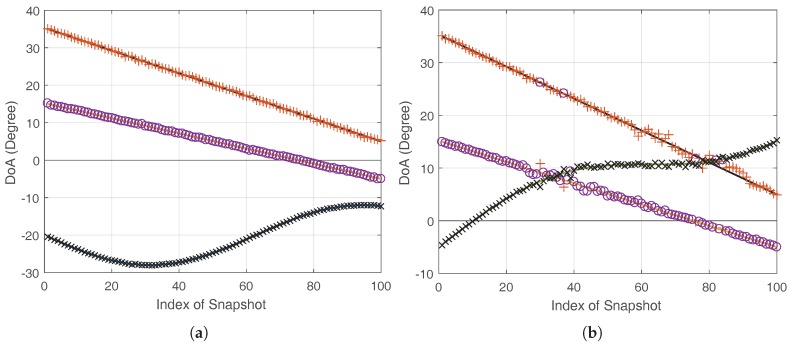
Tracking performance of SIC-SPICE+ with N=20 , K=10 N, and SNR=10 dB. The solid line and markers without line denote actual tracks of sources and estimated DoAs of SIC-SPICE+, respectively. (**a**) Case 1, (**b**) Case 2.

In first experiment, the trajectories estimated by proposed methods are illustrated. The parameter settings are N=20 , K=10 N, and SNR=10 dB. Figure 1 shows estimated trajectories of SIC-SPICE+ in both cases. The estimated trajectories of SIC-SPICE and SIC-SPICE++ are similar and omitted here. In Figure 1a, we can see that SIC-SPICE+ can estimate DoAs correctly. In Figure 1b, fails may happen at cross points. However, by careful observation, it can be found that these fails are caused by the naive data association technique, which does not work at the crossing point. Since data association is out of the scope of this paper, we will focus on Case-1 in the following simulations.

**Figure 2:**
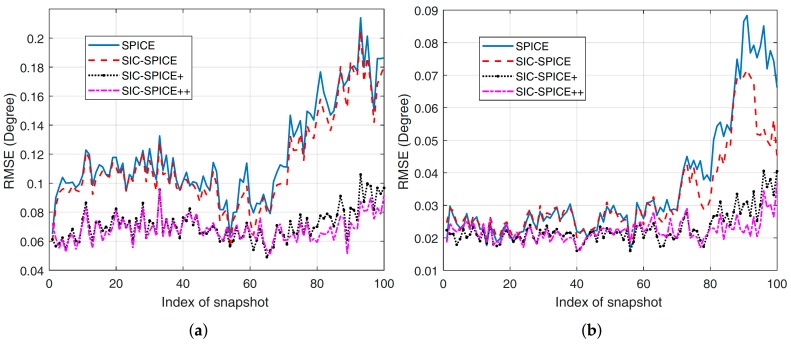
RMSE of different algorithms versus snapshot with N=20 , K=10N , and SNR=20 dB. (**a**) RMSE of the first source (**b**) RMSE of the third source.

The second experiment compares DoA estimation performance of the proposed methods and SPICE in Case-1. The root mean square error (RMSE) versus snapshot index obtained through 50 trials are shown in Figure 2. The RMSE performance of the second source is similar to that of the first one and omitted. We can see that SIC-SPICE outperforms SPICE slightly. The reason is that DoA estimations of both method are not limited by grid points when MUSIC is applied, and off-grid model of SIC-SPICE is more accurate. Moreover, SIC-SPICE+ and SIC-SPICE++ can reduce RMSE significantly. The reason is that SPICE and SIC-SPICE cannot converge to the optimal solution with a small number of iterations, while methods based on moving average have a better initial point and thus converge faster to the optimal point.

**Figure 3:**
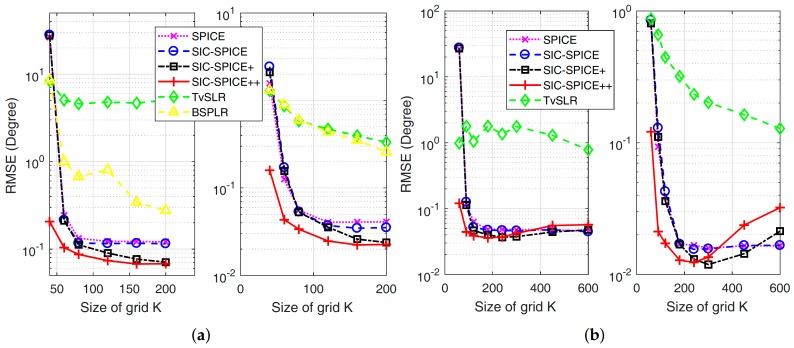
RMSE versus size of gird K with SNR=20 dB for the first (**left**) and third (**right**) sources. (**a**) N=20 , (**b**) N=30 .

The third experiment evaluates performance of the proposed methods with different grid sizes. ULAs with N=20 and N=30 are considered, respectively. The RMSE curves of different algorithms are shown in Figure 3, where each point is based on results of 50 trials. Again, RMSE curve of the second source is omitted. It is seen that TvSLR performs worst. The failure of TvSLR is caused by two reasons: when the SNR is not high enough, e.g., for the first source, TvSLR may suffer from false DoA estimates; when the SNR is large, e.g., for the third source, its RMSE is lower bounded by a number on the same order of $2l_{\epsilon }$ , for the reason that its DoA estimates are restricted by grid points. BSPLR can overcome false estimation, but its estimates are still restricted by the grid. It is seen in Figure 3a that the performance improvement caused by moving average initialization becomes larger as the increase of *K*. The reason is that moving average can provide a more precise information on spatial spectrum based on estimates of the previous outer iteration. Furthermore, SIC-SPICE++ is attractive when *K* is small. This is because the grid of SIC-SPICE++ is more fine around zero and more coarse around $\pm 90^{\circ }$ , and the range of true DoAs is around zero. Although performance loss may be induced if DoAs are large, the effective working area of a ULA is usually in $\left(-60^{\circ },60^{\circ }\right)$ in many practical applications [1]. In Figure 3b, when *K* is too large such that the assumption $|\theta_{m}\left(t\right)-\theta_{m}\left(t-1\right)|\leq 2Bl_{\epsilon }$ with B=1 is not satisfied, moving average leads to performance loss in SIC-SPICE+ and SIC-SPICE++. At this point, B>1 is required, such that this assumption can be satisfied.

**Figure 4:**
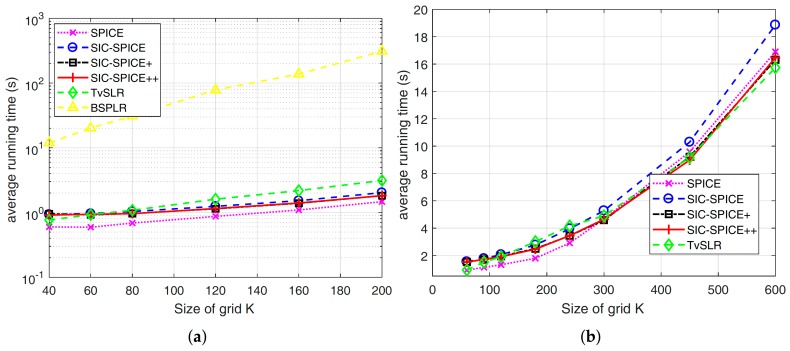
Average running time of different algorithms corresponding to Figure 3. (**a**) N=20 (**b**) N=30 .

In addition, the average running times of different algorithms are plotted in Figure 4. It is seen that BSPLR is time consuming and the average running time of the others are similar. It should be mentioned that when *N* and *K* are both large, e.g., N=90 and K=20 N (with $2l_{\epsilon }=0.1^{\circ }$ ) as in [16], TvSLR is faster than SPICE. Luckily, according to Figure 3, the latter does not require such a large *K* to achieve an accuracy on the order of $0.1^{\circ }$ in a ULA. For other kinds of arrays, such as uniform circular arrays, SIC-SPICE methods, which are based on off-grid models, can achieve a high estimation accuracy with a relatively small *K*.

**Figure 5:**
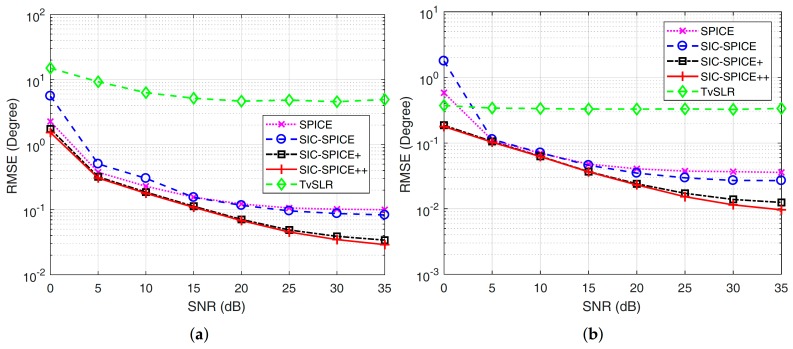
RMSE of different algorithms versus SNR. (**a**) RMSE of the first source (**b**) RMSE of the third source.

The last simulation assesses RMSE of algorithms versus SNR in Case-1 when N=20 and K=10 N. BSPLR is not considered for the heavy workload. RMSE curves of the first and third sources are illustrated in Figure 5. It is seen that TvSLR is lower bounded, for the reason of error DoA estimation as well as on grid constraint. SPICE is better than SIC-SPICE at the low SNR region, the reason is that SIC-SPICE is not converged. Note that the number of inner iterations is $I_{1}=3$ in SIC-SPICE and 6 in SPICE. As SNR increases, SIC-SPICE outperforms SPICE, since the estimation error is dominated by modeling errors at the moment. SIC-SPICE+ and SIC-SPICE++ outperform the others, especially at the high SNR region.

## 5. Conclusions

In this paper, DoA estimation and tracking algorithms are discussed in the framework of SSR. The proposed methods are extensions and improvements of C-SPICE, which is an efficient off-grid method for estimating constant DoAs. Numerical experiments demonstrate the validity and efficiency of the proposed methods. An accurate data association algorithm under the framework of SSR can be investigated in the future.

## Appendix A

First, define vector $c_{k}\in C^{N\times 1}$ and matrix $C_{\sigma }\in C^{N}$ and consider the following problem

(A1)
$$\begin{array}{cc} \min_{c_{k},C_{\sigma }}\; & \sum_{k=1}^{K}\frac{1}{p_{k}}∥c_{k}{∥}^{2}+\frac{1}{\sigma }{∥C_{\sigma }∥}^{2},\;s.t.\;\sum_{k=1}^{K}g_{k}c_{k}^{H}+C_{\sigma }={\hat{R}}^{0.5}. \end{array}$$

Making use of Lagrange multiplier will show that the optimal solution of (A1) is

(A2)
$$\begin{array}{c} c_{k}=p_{k}{\hat{R}}^{0.5}R_{\hat{\epsilon }}^{-1}g_{k},\;\;C_{\sigma }=\sigma R_{\hat{\epsilon }}^{-1}{\hat{R}}^{0.5} \end{array}$$

and the corresponding optimal value is $\mathrm{tr}(\hat{R}R_{\hat{\epsilon }}^{-1})$ , which is the first term of objective function of problem (13). As a result, the optimal solution of (13) can be obtained by solving

(A3a)minpk≥0,σ≥0,ck,Cσfe(pk,σ,ck,Cσ)(A3b)s.t.fe(pk,σ,ck,Cσ)=∑k=1K1pk∥ck∥2+1σ∥Cσ∥2+tr(R^−1Rϵ^),(A3c)∑k=1KgkckH+Cσ=R^0.5.

In problem (A3), $f_{e}\left(p_{k},\sigma ,c_{k},C_{\sigma }\right)$ can be equivalently cast into $f_{e}\left(p_{k},\sigma ,c_{k},C_{\sigma }\right)=\sum_{k=1}^{K}t_{k}+t_{\sigma }+\mathrm{tr}\left({\hat{R}}^{-1}R_{\hat{\epsilon }}\right)$ with hyperbolic constraints $t_{k}p_{k}\geq {∥c_{k}∥}^{2}$ and $\sigma t_{\sigma }\geq {∥C_{\sigma }∥}^{2}$ . Therefore, problem (A3) is actually a second order cone program [22] and thus convex. Hence, it can be optimally solved by updating $\left(c_{k},C_{\sigma }\right)$ and $\left(p_{k},\sigma \right)$ iteratively.

When $\left(p_{k},\sigma \right)$ is given, $\left(c_{k},C_{\sigma }\right)$ is updated by (A2). When $\left(c_{k},C_{\sigma }\right)$ is given and based on the inequality of arithmetic and geometric means, we have

(A4)
$$\begin{array}{cc} f_{e}\left(p_{k},\sigma ,c_{k},C_{\sigma }\right) & =\sum_{k=1}^{K}\frac{1}{p_{k}}∥c_{k}{∥}^{2}+\frac{1}{\sigma }{∥C_{\sigma }∥}^{2}+\sum_{k=1}^{K}p_{k}g_{k}^{H}{\hat{R}}^{-1}g_{k}+\sigma \mathrm{tr}\left({\hat{R}}^{-1}\right)\\ & \geq \sum_{k=1}^{K}∥c_{k}∥\sqrt{g_{k}^{H}{\hat{R}}^{-1}g_{k}}+∥C_{\sigma }∥\sqrt{\mathrm{tr}({\hat{R}}^{-1})}, \end{array}$$

and equation holds when $p_{k}=∥c_{k}∥/\sqrt{g_{k}^{H}{\hat{R}}^{-1}g_{k}}$ and $\sigma =∥C_{\sigma }∥/\sqrt{\mathrm{tr}({\hat{R}}^{-1})}$ . Omitting the updating of $\left(c_{k},C_{\sigma }\right)$ , we have

(A5)
$$\begin{array}{c} p_{k}={\hat{p}}_{k}∥{\hat{R}}^{0.5}R_{\hat{\epsilon }}^{-1}g_{k}∥/∥{\hat{R}}^{-0.5}g_{k}∥,\;k\in \left[K\right],\;\sigma =\hat{\sigma }∥R_{\hat{\epsilon }}^{-1}{\hat{R}}^{0.5}∥/\sqrt{\mathrm{tr}({\hat{R}}^{-1})}, \end{array}$$

where ${\hat{p}}_{k}$ and $\hat{\sigma }$ denote estimates of $p_{k}$ and σ in the previous iteration, respectively, and $R_{\hat{\epsilon }}$ is calculated based on ${\hat{p}}_{k}$ and $\hat{\sigma }$ .

Since problem (A3) is convex, its optimal solution can be attained from any initial point in the interior of feasible set, one of which is provided by the periodogram (PER) method [14], i.e.,

(A6)
$$\begin{array}{cc} p_{k} & ={∥{\hat{R}}^{0.5}g_{k}∥}^{2}/{∥{\hat{R}}^{-0.5}g_{k}∥}^{4},\;\;\forall k\in \left[K\right],\;\sigma =\frac{1}{N}\sum_{s=1}^{N}p_{k_{s}}{∥g_{k_{s}}∥}^{2}, \end{array}$$

where ${\left\{k_{s}\right\}}_{s=1}^{N}$ denotes the index set of *N* smallest $p_{k}$ .
